# Multi-Omics Data Integration Analysis of an Immune-Related Gene Signature in LGG Patients With Epilepsy

**DOI:** 10.3389/fcell.2021.686909

**Published:** 2021-07-16

**Authors:** Quan Cheng, Weiwei Duan, Shiqing He, Chen Li, Hui Cao, Kun Liu, Weijie Ye, Bo Yuan, Zhiwei Xia

**Affiliations:** ^1^Department of Neurosurgery, Xiangya Hospital, Central South University, Changsha, China; ^2^National Clinical Research Center for Geriatric Disorders, Xiangya Hospital, Central South University, Changsha, China; ^3^Department of Neurology, Xiangya Hospital, Central South University, Changsha, China; ^4^Department of Neurosurgery, Affiliated Nanhua Hospital, Hengyang Medical College, University of South China, Hengyang, China; ^5^Department of Rehabilitation Medicine, Hunan Provincial People’s Hospital, Hunan Normal University, Changsha, China; ^6^Department of Psychiatry, The Second People’s Hospital of Hunan Province, The Hospital of Hunan University of Chinese Medicine, Changsha, China; ^7^Department of Cerebrovascular Surgery, The Second People’s Hospital of Hunan Province, The Hospital of Hunan University of Chinese Medicine, Changsha, China; ^8^Department of Clinical Pharmacology, Xiangya Hospital, Central South University, Changsha, China; ^9^Department of Neurology, Hunan Aerospace Hospital, Changsha Medical University, Changsha, China

**Keywords:** glioma, epilepsy, immune, immunotherapy, prognosis

## Abstract

**Background:**

The tumor immune microenvironment significantly affects tumor occurrence, progression, and prognosis, but its impact on the prognosis of low-grade glioma (LGG) patients with epilepsy has not been reported. Hence, the purpose of this study is to explore its effect on LGG patients with epilepsy.

**Methods:**

The data of LGG patients derived from the TCGA database. The level of immune cell infiltration and the proportion of 22 immune cells were evaluated by ESTIMATE and CIBERSORT algorithms, respectively. The Cox and LASSO regression analysis was adopted to determine the DEGs, and further established the clustering and risk score models. The association between genomic alterations and risk score was investigated using CNV and somatic mutation data. GSVA was adopted to identify the immunological pathways, immune infiltration and inflammatory profiles related to the signature genes. The Tumor Immune Dysfunction and Exclusion (TIDE) algorithm and GDSC database were used to predict the patient’s response to immunotherapy and chemotherapy, respectively.

**Results:**

The prognosis of LGG patients with epilepsy was associated with the immune score. Three prognostic DEGs (ABCC3, PDPN, and INA) were screened out. The expression of signature genes was regulated by DNA methylation. The clustering and risk score models could stratify glioma patients into distinct prognosis groups. The risk score was an independent predictor in prognosis, with a high risk-score indicating a poor prognosis, more malignant clinicopathological and genomic aberration features. The nomogram had the better predictive ability. Patients at high risk had a higher level of macrophage infiltration and increased inflammatory activities associated with T cells and macrophages. While the higher percentage of NK CD56bright cell and more active inflammatory activity associated with B cell were present in the low-risk patients. The signature genes participated in the regulation of immune-related pathways, such as IL6-JAK-STAT3 signaling, IFN-α response, IFN-γ response, and TNFA-signaling-via-NFKB pathways. The high-risk patients were more likely to benefit from anti-PD1 and temozolomide (TMZ) treatment.

**Conclusion:**

An immune-related gene signature was established based on ABCC3, PDPN, and INA, which can be used to predict the prognosis, immune infiltration status, immunotherapy and chemotherapy response of LGG patients with epilepsy.

## Introduction

As the most common brain malignant tumor, patients with glioma are often complicated with epilepsy (Goldstein and Feyissa, 2018; Jones et al., 2019; Liu B. et al., 2019). Compared with high-grade glioma, patients with low-grade glioma (LGG) have a significantly higher incidence of epilepsy (Huberfeld and Vecht, 2016), which may be related to the slower growth rate and higher IDH-1 mutation frequency of LGG (Cohen et al., 2013; Chen et al., 2018). The effect of epilepsy on the prognosis of LGG is currently unclear. Several studies have suggested that epilepsy is a positive prognostic factor in LGG patients (Blümcke et al., 2004; Krzyszkowski et al., 2004; Danfors et al., 2009). However, Lote et al. (1998) found that epilepsy had no effect on the prognosis after analyzing 379 LGG patients. This indicates that there are other factors affecting the prognosis of LGG patients with epilepsy.

In addition to tumor cells, non-tumor cells including immune cells are also important components of tumors. Immune cells infiltrating tumor tissues, such as peripheral macrophages, microglia, granulocytes, T lymphocytes, and myeloid-derived suppressor cells (Gieryng et al., 2017), create a tumor immune microenvironment that affects tumor development, metastasis, and response to treatment (Ma et al., 2018). However, its influence on LGG patients with epilepsy has not been established.

Hence, we explored the immune microenvironment of LGG patients with epilepsy and evaluated its impact on prognosis through the integration analysis of multi-omics data. We developed a gene signature related to immune infiltration, which can be used to predict the prognosis and therapeutic response of LGG patients with epilepsy.

## Materials and Methods

### Data Acquisition

The RNA transcript data (Workflow Type: HTSeq-Counts), corresponding clinical information, DNA methylation (Platform: Illumina human methylation 450), somatic mutation (Workflow Type: MuTect2), and copy number variation (CNV) data of 473 LGG patients (WHO grade II and grade III) were downloaded from the TCGA database^1^. Among them, there were 297 patients with epilepsy and 176 patients without epilepsy. The sample screening process was shown in Supplementary Figure 1.

### Inference of Immune Infiltration Level and Immune Cell Abundance

The immune score was calculated by the “ESTIMATE” algorithm (version 1.0.13) (Yoshihara et al., 2013) to infer the level of immune infiltration. The proportions of 22 immune cells were inferred by the “CIBERSORT” algorithm (Newman et al., 2015) using LM22, a leukocyte gene signature matrix^2^.

### Screening of Prognostic Signature Genes

The “DESeq2” R package (version 1.30.1) (Love et al., 2014) was used to search for differentially expressed genes (DEGs), |log(2) fold change| >2 and *p* < 0.05 as a threshold. The Cox regression analysis was adopted to find the DEGs with independent prognostic value. Finally, the DEGs with the most prognostic value and their regression coefficients (β) were obtained by the LASSO regression analysis.

### Consensus Clustering Analysis

The consensus clustering analysis was conducted using the “ConsensusClusterPlus” R package (version 1.54.0) (Wilkerson and Hayes, 2010). The clustering run was permuted by varying the category number k from 2 to 10. The optimal *k* value was determined based on good consistency within the clusters and a relatively small incremental change in the area under the CDF curve.

### Establishment and Evaluation of Risk Score Model

The risk score model was established as follows: $r\,i\,s\,k\,s\,c\,o\,r\,e=\Sigma_{i=1}^{n}\,\left({\text{Geneexpr}}_{i}\times \beta_{i}\right)$. The effectiveness of the risk model was evaluated by comparing the prognosis of high- and low-risk patients and calculating the AUC value of the ROC curve. The correlation between risk score and clinicopathological characteristics was further analyzed. The R package “rms” (version 6.1-0) was used to construct a nomogram.

### Analysis of Genetic Alterations

The somatic mutation data were processed by the “maftools” R package (version 2.6.05) (Mayakonda et al., 2018), and the genes with the highest mutation frequency were screened and presented. Genomic Identification of Significant Targets in Cancer (GISTIC) 2.0 (Mermel et al., 2011) was used to analyze CNV data in reference to the Human genome reference consortium h19 derived from GISTIC 2⋅0. The threshold copy number at alteration peaks was obtained from GISTIC analysis.

### The GSVA Analysis

The gene set variation analysis (GSVA) (Hänzelmann et al., 2013) was used to establish each sample’s immune signature and the correlation analysis was further carried out. The correlogram and heatmap were drawn using the “corrgram” (version 1.13) and “pheatmap” R packages (version 1.0.12), respectively. The immunological pathways associated with signature genes were identified by GSVA based on the hallmark gene sets (Liberzon et al., 2015).

### Therapy Response Prediction

The patients’ response to immune checkpoint blocking therapy was predicted by the Tumor Immune Dysfunction and Exclusion (TIDE) (Jiang et al., 2018) algorithm. The patients’ sensitivity to temozolomide (TMZ) therapy was predicted using the data derived from the GDSC database^3^. The “pRRophetic” R package (version 0.5) (Geeleher et al., 2014) was used for the estimation of the half-maximal inhibitory concentration (IC50), which represents the drug response.

### Statistical Analysis

The statistical analysis was finished in R software (version 3.5.3). Statistical significance between groups was determined using the Student’s *t*-test. Pearson rank was adopted in the correlation analysis. The “survival” R package (version 3.2-7) and Kaplan-Meier method were used for survival analysis. The AUC value of ROC curve was calculated by the “survival ROC” R package (version 1.0.3). *P* < 0.05 was considered to be statistically significant.

## Results

### The Level of Immune Infiltration Affected the Prognosis

**FIGURE 1:**
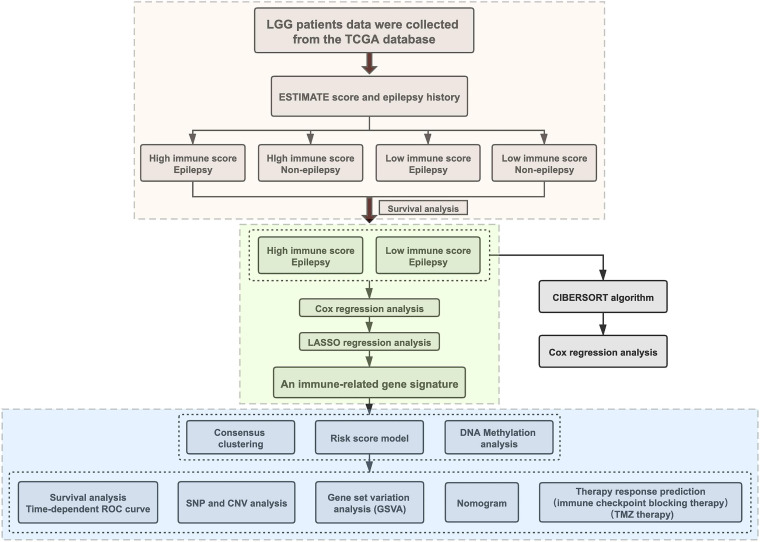
The flowchart designed for the study.

**FIGURE 2:**
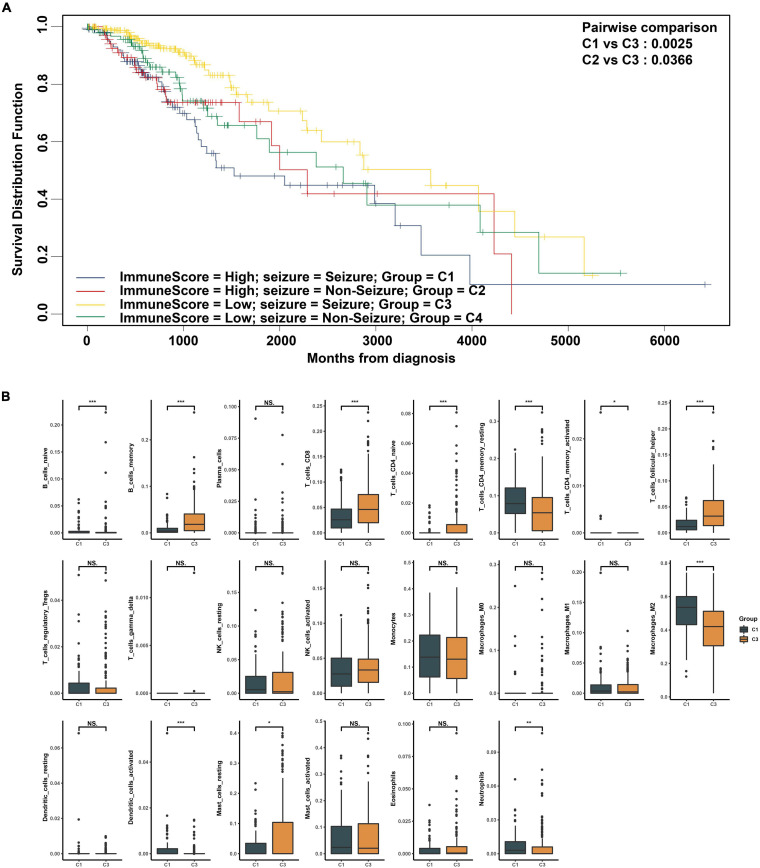
**(A)** Comparison of survival rate of low-grade glioma (LGG) subgroups differentiated by seizure and immune score. **(B)** Comparison of 22 immune cell components between high and low immune score groups in LGG patients with epilepsy. *p < 0.05, **p < 0.01, ***p < 0.001.

The characteristics of all included patients were summarized in Supplementary Table 1. The workflow designed for the study was shown in Figure 1. According to the immune score and whether they had epilepsy, all patients were divided into four groups: C1(high immune score, seizure), C2 (high immune score, non-seizure), C3 (low immune score, seizure), and C4 (low immune score, non-seizure). Here, the immune scores that were >0 were considered high while immune scores that were <0 were considered low (Wen et al., 2020). Through survival analysis, we found that a high immune score suggested a poor prognosis in LGG patients with epilepsy (*p* = 0.0025, Figure 2A). The baseline characteristics of LGG patients with epilepsy were shown in Supplementary Table 2. We further compared the abundance of 22 immune cells between high and low immune score groups in LGG patients with epilepsy. The results showed that the patients with high immune score had a higher percentage of macrophages (M2), and a higher proportion of naive B cells, activated dendritic cells, neutrophils, activated memory CD4^+^ T cells, and resting memory CD4^+^ T cells also appeared in the high immune score group (*p* < 0.05, Figure 2B). While the patients in the low immune score group had higher percentages of memory B cells, naive CD4^+^ T cells, resting mast cells, CD8^+^ T cells, and follicular T helper cells (*p* < 0.05, Figure 2B). Further Cox regression analysis indicated that activated memory CD4^+^ T cells and activated dendritic cells were negative factors for prognosis (*p* < 0.05, Supplementary Table 3).

### Three Prognostic Signature Genes Were Identified

**FIGURE 3:**
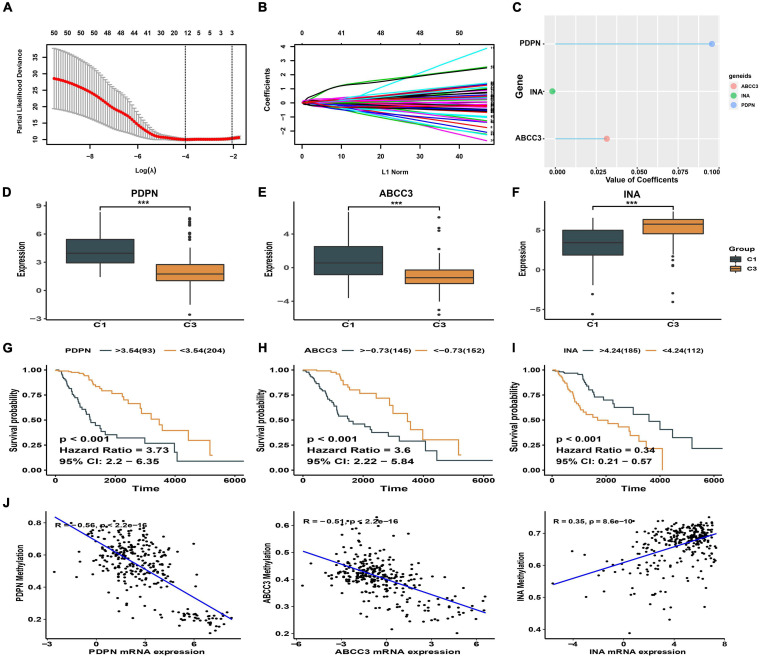
Identification of signature genes. **(A)** The cross-validation plot of LASSO regression [the dashed lines signify the optimal log (λ) value]. **(B)** LASSO coefficients of 51 DEGs with independent prognostic value. **(C)** LASSO coefficients of the signature genes. **(D–F)** Expression difference of ABCC3, PDPN, INA between high and low immune score groups. **(G–I)** Kaplan-Meier plot showing different prognosis based on the differential expression of ABCC3, PDPN, and INA. **(J)** Correlation between DNA methylation and signature genes expression. ***p < 0.001.

A total of 12278 DEGs were found between the high and low immune score groups in LGG patients with epilepsy. Furthermore, 51 DEGs with independent prognostic value were identified by Cox regression analysis (*p* < 0.05). Finally, three genes (ABCC3, INA, PDPN) with the most prognostic value were obtained using the LASSO regression (Figures 3A,B) and their coefficients (ABCC3: 0.0314, INA: −0.0018, PDPN: 0.0957) were shown in Figure 3C. Consequently, a prognostic immune-related gene signature was established. We found that the high immune score group had higher expression levels of ABCC3 and PDPN, while the low immune score group showed higher expression of INA (*p* < 0.001, Figures 3D–F). The high expression of ABCC3 (HR 3.6, *p* < 0.001) and PDPN (HR 3.73, *p* < 0.001) was associated with poor prognosis (Figures 3G,H), while the high expression of the INA (HR 0.34, *p* < 0.001) was a positive prognostic factor (Figure 3I). This indicates that PDPN and ABCC3 are risk genes, and INA is a protective gene for LGG patients with epilepsy.

We also explored the relationship between the methylation levels of signature genes and their expression levels in LGG patients with epilepsy. We found that the expression of ABCC3 (*R* = −0.51, *p* < 0.001) and PDPN (*R* = −0.56, *p* < 0.001) genes was negatively correlated with methylation levels (*p* < 0.001), while the INA (*R* = 0.35, *p* < 0.001) was positively correlated (Figure 3J and Supplementary Figure 2). This suggests that the hypomethylation of ABCC3 and PDPN is present in LGG patients with epilepsy who have high immune score and poor prognosis.

### Clustering Model Based on Signature Genes Identified Two Subgroups With Distinct Outcomes

The samples of LGG patients with epilepsy were grouped using consensus clustering analysis to preliminarily explore the prognostic value of signature genes. In consensus clustering analysis, *k* = 2 was determined as the optimal selection, which had the small incremental change in the area under CDF curve while keeping the maximal consensus within clusters (Supplementary Figures 3A–C). Therefore, we could obtain two patient subgroups, cluster1 and cluster2. And the principal component analysis (PCA) could obviously distinguish the two clusters, which also proved the rationality of the clustering (Supplementary Figure 3D). A significant difference in prognosis was found between cluster1 and cluster2. The overall survival (OS), disease specific survival (DSS), and progression free interval (PFI) of patients in cluster1 were all better than those in cluster 2 (*p* < 0.0001, Supplementary Figure 3E).

### Risk Score Model Based on Signature Genes Had Excellent Predictive Ability in Prognosis

**FIGURE 4:**
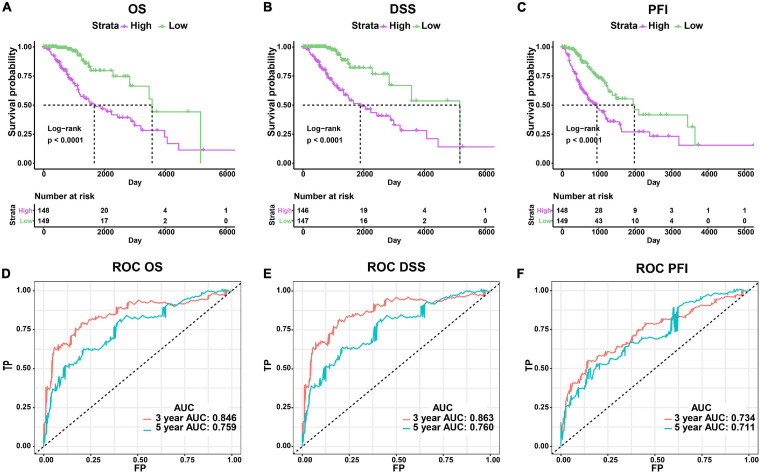
Evaluation of the risk score model. **(A–C)** Comparison of clinical outcomes between high- and low-risk groups using OS, DSS, and PFI as the endpoints. **(D–F)** ROC curves and AUC values of risk model.

We established a risk score model based on the signature genes. According to the median value of patients’ risk score calculated by the risk score model, the LGG patients with epilepsy were divided into high- and low-risk groups. The prognosis of patients was significantly different between the two groups, and the OS, DSS, and PFI of patients with high risk were significantly worse (*p* < 0.0001, Figures 4A–C). The AUC values of 3-year and 5-year ROC curves of the risk model were 0.846 and 0.759 in OS, 0.863 and 0.760 in DSS, 0.734 and 0.711 in PFI, respectively (Figures 4D–F). The above results show that the risk model has a good prognosis prediction ability.

### Risk Score Was Correlated With Clinicopathological and Genomic Aberration Features

We conducted a subgroup analysis to explore the correlation between clinicopathological features and risk score. We found significant differences in risk scores within the subgroups distinguished by MGMT promoter status, IDH status, subtype, WHO grade, and 1p19q codel status (*p* < 0.001), while no significant differences were found within gender and age subgroups (*p* > 0.05). Patients with unmethylated MGMT promoter, 1p/19q non-codel, classical, and mesenchymal subtype, IDH-wildtype, and higher WHO grade had higher risk scores (Supplementary Figure 4).

**FIGURE 5:**
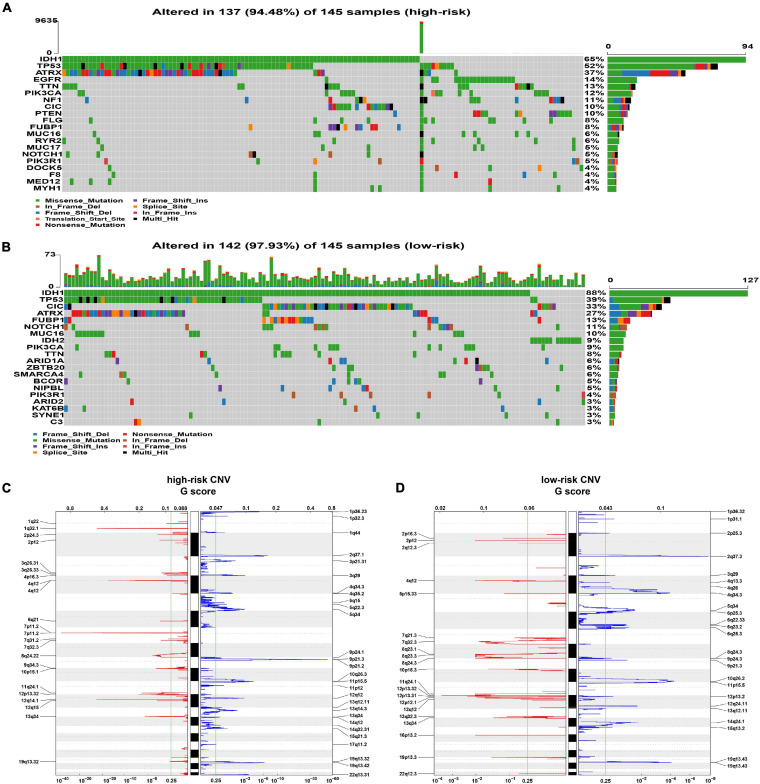
Analysis of genomic alterations. **(A,B)** The somatic mutation profiles in high-risk (A) and low-risk (B) groups. **(C,D)** The analysis of CNV. The deleted (blue) and amplified (red) chromosomal regions in high- panel **(C)** and low-risk panel **(D)** groups; *q* value = 0.25 as the threshold (the green line).

Somatic mutations were observed in 137 (94.48%) of the high-risk samples and in 142 (97.93%) samples of the low-risk group. The mutation frequency of IDH1, CIC, and IDH2 genes in high-risk patients was lower than that in low-risk patients (IDH1: 65% vs. 88%, *p* < 0.001; CIC: 10% vs. 33%, *p* < 0.001; IDH2 0% vs. 9%, *p* < 0.001), while high-risk patients had a higher frequency of mutations in EGFR, NF1, and PTEN (EGFR: 14% vs. 0%, *p* < 0.001; NF1: 11% vs. 1%, *p* < 0.001; PTEN 10% vs. 1%, *p* < 0.001) (Figures 5A,B and Supplementary Figure 5).

In the analysis of CNV data, several significant amplification regions containing multiple oncogenes, such as 12q14.1(CDK4), 7p11.2 (EGFR), 4q12 (PDGFRA), and 1q32.1 (PIK3C2B), were identified in high-risk patients (Figure 5C). In addition, focal deletion peak 9p21.3 (CDKN2A, CDKN2B) was detected in patients with high risk (Figure 5C). In the low-risk group, the focal amplification and deletion peaks also appeared, but they had significantly lower *G* values (Figure 5D).

### The Risk Score Was an Independent Predictor for Prognosis

**FIGURE 6:**
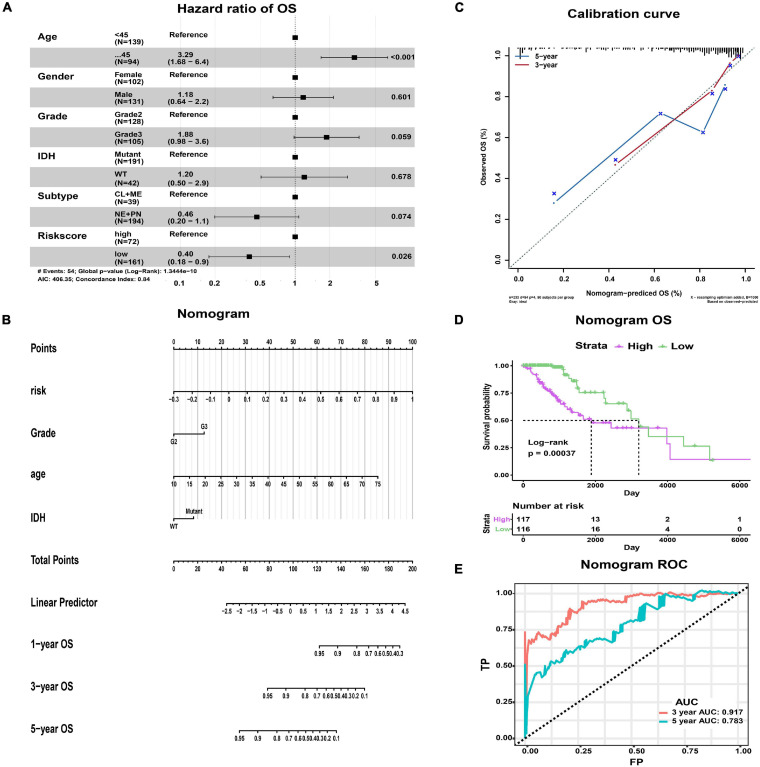
The nomogram predicting the OS of LGG patients with epilepsy. **(A)** Forest map showing the results of multivariate Cox regression analysis. **(B)** The nomogram was used by summing the points of each variable. **(C)** The calibration curves showing the predicted and actual observed OS rates. **(D)** K-M plot showing the difference in OS between high- and low-risk groups. **(E)** The ROC curves and AUC values of the nomogram.

We comprehensively analyzed the effect of risk score and clinicopathological characteristics on a clinical outcome to assess whether the risk score has an independent prognostic value. In univariate Cox regression analysis, age, WHO grade, IDH status, subtype, and risk score were identified as survival-related factors (*p* < 0.001, Supplementary Table 4). Further multivariate Cox regression analysis indicated that age and risk score were independent prognostic factors. Age ≥ 45 (HR 3.29, *p* < 0.001) was associated with a worse prognosis, while low risk score (HR 0.40, *p* = 0.026) was a benign prognostic factor (Figure 6A).

In addition, we constructed a nomogram that integrated the clinicopathological features and risk score to improve clinical feasibility (Figure 6B). The predicted 3/5-year OS were close to the actually observed ones, as shown in the calibration curve (Figure 6C). According to the median value of patients’ points calculated by the nomogram, we could also obtain two groups of patients, the high-risk group and the low-risk group. Survival analysis suggested that high-risk patients had worse OS than low-risk patients (*p* = 0.00037, Figure 6D). The AUC values of ROC curves from the nomogram were 0.917 at predicted 3-year OS and 0.783 at 5-year OS, both of which were better than those obtained by using the risk model alone (Figure 6E). Therefore, the nomogram has better predictive power compared with the risk model.

### Immunological Pathways, Immune Infiltration and Inflammatory Profiles Related to Signature Genes

**FIGURE 7:**
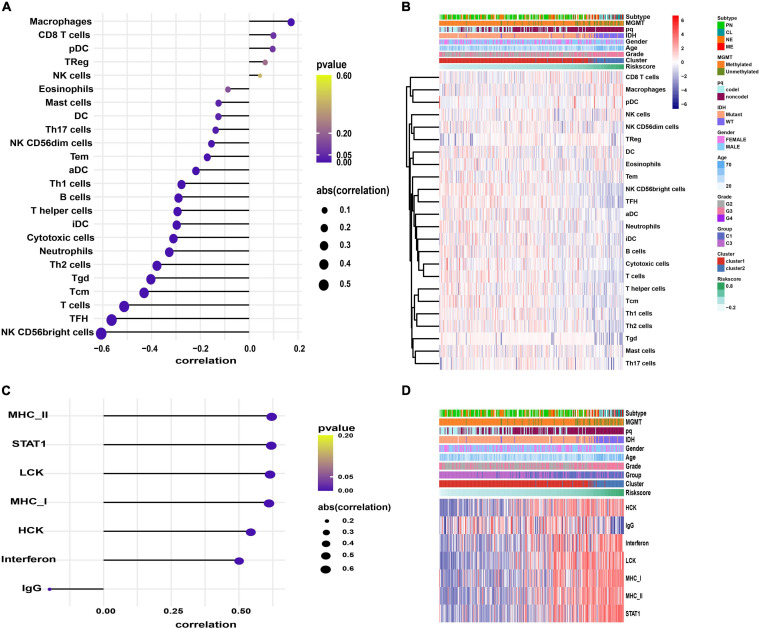
The GSVA results. **(A,C)** Correlograms showing the correlation between immune cell infiltration, inflammation activities, and risk score. **(B,D)** Heatmaps displaying the activities of immune cells panel **(B)** and inflammation panel **(D)**.

We explored the immunological activities related to the signature genes. We first carried out the GSVA based on specific marker genes of 24 immune cells and seven metagenes representing different types of inflammation and immune responses (Rody et al., 2009). Further correlation analysis showed that macrophages infiltration was positively correlated with risk score (*p* = 0.002; Figures 7A,B and Supplementary Table 5). While B cells, T cells, T helper (Th) cells, follicular helper T cell (TFH), Th17 cells, Th1 cells, Th2 cells, gamma delta T Cells (Tgd), central memory T cell (Tcm), effector memory T cell (Tem), NK CD56dim cells, NK CD56bright cells, mast cells, dendritic cell (DC), activated DC (aDC), immature DC (iDC), cytotoxic cells, and neutrophils infiltration were negatively correlated with risk score, especially the NK CD56bright cells (*p* < 0.05; Figures 7A,B and Supplementary Table 5). In terms of inflammatory activities, MHC-II, STAT1, LCK, MHC-I, HCK, and Interferon were positively correlated with risk score, while IgG, a marker for B cells, was negatively correlated (*p* < 0.001; Figures 7C,D and Supplementary Table 6).

We also conducted GSVA to identify the immunological pathways associated with the signature genes. Compared with the low-risk group, the IL6-JAK-STAT3 signaling, IFN-α response, IFN-γ response, and TNFA-signaling-via-NFKB pathways were significantly active in patients with high-risk (*p* < 0.05, Supplementary Figure 6).

### Risk Score Was Correlated With Immunotherapy and Chemotherapy Response

**FIGURE 8:**
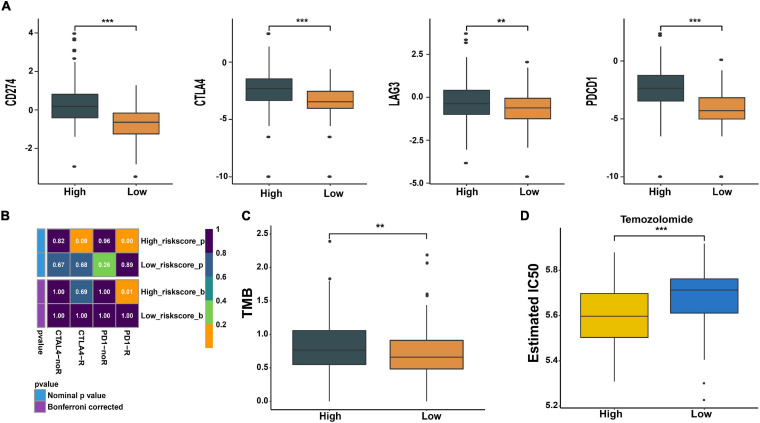
Prediction of response to immunotherapy and chemotherapy. **(A)** Expression of immune checkpoints in high- and low-risk groups. **(B)** The analysis of response to immunotherapy. **(C)** Comparison of TMB between the two groups. **(D)** The estimated IC50 value of temozolomide (TMZ) in the two groups. **p < 0.01, ***p < 0.001.

Cancer growth and progression are associated with immunosuppression, and it is currently believed that the immune checkpoint pathways are the major mechanism of tumor immune resistance (Pardoll, 2012). The immune checkpoint blockade has become a promising immunotherapy method for various tumors including glioma (Pardoll, 2012; Hodges et al., 2017). In this study, we found that immune checkpoint molecules CD724 (PD-L1), CTL4, LAG3, and PDCD1 (PD1) were expressed at higher levels in high-risk patients (*p* < 0.01, Figure 8A). We further predicted the patients’ response to anti-CTLA4 and anti-PD1 therapy and found that high-risk patients were more likely to respond to anti-PD1 therapy than low-risk patients (*p* = 0.01, Figure 8B). No significant difference was observed in response to anti-CTLA4 therapy between the two groups (*p* > 0.05, Figure 8B). Moreover, a higher tumor mutation burden (TMB) was observed in high-risk patients (*p* < 0.01, Figure 8C).

Meanwhile, we also predicted the patients’ sensitivity to TMZ therapy, which is currently the most commonly used chemotherapeutics for glioma. We trained a predictive model using cell line data derived from the GDSC database. With this predictive model, the IC50 value of each patient’s TMZ was estimated. We found that the estimated IC50 value in low-risk patients was higher compared with high-risk patients (*p* < 0.001, Figure 8D). This indicates that patients with high risk may be more sensitive to TMZ therapy.

## Discussion

In this study, we explored the effect of the immune microenvironment on the prognosis of LGG patients with epilepsy. We found that the immune score was associated with survival, and patients with a high immune score had a poor prognosis. Moreover, the difference of immune cell infiltration between different immune score groups was observed, and a higher proportion of M2 macrophages appeared in high immune score patients. The M2 macrophage infiltration subverts the host’s adaptive immune response and fosters a tumor milieu ripe for angiogenesis, migration, and metastasis in the glioma (Michelson et al., 2016; Liu et al., 2018). These findings indicate that the immune microenvironment affects the prognosis of LGG patients with epilepsy.

Further, three prognostic DEGs (ABCC3, INA, and PDPN) were identified between high and low immune score groups in LGG patients with epilepsy. Hence, a prognostic immune-related gene signature was established. The ATP binding cassette subfamily C member 3 (ABCC3) gene belongs to the ABCC gene family encoding the ABC transporters and participates in transporting ATP-dependent substances across lipid membranes (Chen and Tiwari, 2011; Mao et al., 2019). A high expression level of the ABCC3 gene is associated with various tumors’ poor prognosis, such as gastric cancer, breast cancer, and pancreatic cancer (Adamska et al., 2019; Mao et al., 2019; Wang et al., 2020). Podoplanin (PDPN) is a glycoprotein receptor that affects cell behavior by interacting with other proteins and has various physiological functions (Krishnan et al., 2018). The PDPN is crucial for the development of the lymphatic system, lungs, and heart (Astarita et al., 2012). It is also a malignant tumor promoter, which promotes tumor migration, invasion, lymphangiogenesis, and angiogenesis (Astarita et al., 2012; Quintanilla et al., 2019); Its up-regulation in tumor tissues is correlated with the poor prognosis of mesothelioma, melanoma, and squamous cell carcinoma (Watanabe et al., 2020). Alpha-internexin (INA) gene encodes a neurofilament interacting protein and is involved in neuronal development and axonal outgrowth (Ducray et al., 2011; Rajmohan et al., 2020). The reduced/loss of its expression is related to tumor invasion and suggests a poor prognosis in patients with gastroenteropancreatic neuroendocrine neoplasms (GEP-NENs) (Wang et al., 2018). In this study, the high expression of ABCC3 and PDPN genes was found in the high immune score group and was related to the poor prognosis of LGG patients with epilepsy. And the high expression of INA gene was observed in patients with low immune score and suggests a better prognosis. We also found that the expression level of signature genes was regulated by DNA methylation in LGG patients with epilepsy. DNA methylation is associated with gene silencing and tumorigenesis, and tumors are often accompanied by hypomethylation of oncogenes and hypermethylation of tumor suppressor genes (Morgan et al., 2018). This also provides new potential targets for the regulation of glioma epigenetics.

We established a risk score model based on the above signature genes, and the high risk-score suggested a poor prognosis. The risk score was determined as a factor with an independent prognostic value. The 1p/19q non−codel, IDH-wildtype, classical and mesenchymal subtype, MGMT promotor unmethylated, and higher WHO grade, which are the malignant clinicopathological features in glioma (Verhaak et al., 2010; Weller et al., 2012), were associated with a higher risk score. In addition, the frequency of IDH1, CIC, and IDH2 mutations suggested a positive prognosis (Appin and Brat, 2014; Miller et al., 2017; Liu Z. et al., 2019) were lower in high-risk patients, while the malignancy driving mutations (EGFR, NF1, and PTEN) (Appin and Brat, 2014; Asif et al., 2019) were more frequent. And the deletion peaks of tumor suppressor genes (CDKN2A, CDKN2B) and amplification peaks of oncogenes (EGFR, CDK4, PDGFRA, PIK3C2B) (Rao et al., 2010) were also detected in patients with high risk. These results suggest that this gene signature is a reliable prognostic predictor for LGG patients with epilepsy. Additionally, the nomogram constructed by integrating the clinicopathological features and risk score had a better prognostic prediction ability, and further improves the clinical practicality.

The relationship between the immune microenvironment and gene signature was further explored. The high- and low-risk patients had different immune cell infiltration and inflammatory profiles. Macrophage infiltration level was higher in high-risk patients, while low-risk patients had higher B cells, T cells, Tgd, Th1 cells, T helper cells, Th2 cells, TFH, Tem, Tcm, Th17 cells, NK CD56bright cells, NK CD56dim cells, mast cells, iDC, aDC, DC, cytotoxic cells, and neutrophils infiltration, especially the NK CD56bright cells. Macrophages are recruited to the glioma environment and create a supportive stroma for neoplastic cell invasion and expansion through the release of various growth factors and cytokines, and the number of macrophages increases with increasing malignancy grade (Hambardzumyan et al., 2016). The CD56bright cells are a functional subset of NK cells, which are characterized by low cytotoxicity and high cytokine production, and play an immunoregulatory role (Shevtsov and Multhoff, 2016). The infiltration of NK CD56bright cells is negatively correlated with the malignant degree of gastric carcinomas, lung tumors, and papillary thyroid cancer (Gogali et al., 2013). However, the effect of NK CD56bright cells on glioma has not been sufficiently explored. The infusion of IL15, which is required for survival, expansion, and activation of NK cells, significantly expands the subpopulation of CD56 bright NK Cells compared with the CD56dim NK Cells (Dubois et al., 2017), another functional subset of NK cells with high cytotoxic activity and low cytokine release properties (Shevtsov and Multhoff, 2016). Moreover, infusion of IL-15 in glioma model mice significantly increases the infiltration of NK cells into tumor and reduces tumor growth (Garofalo et al., 2015). Combined with the results of this study, we believe that NK CD56bright cells play an important role in glioma immunity, and its infiltration improves the prognosis. In addition, the high-risk patients had more active inflammatory responses related to T cells and macrophages, while patients with low risk had more active inflammatory response associated with B cells. We further explored the mechanism of signature genes involved in the regulation of the immune microenvironment, and found that the signature genes participated in the regulation of IL6-JAK-STAT3 signaling, IFN-α response, IFN-γ response, and TNFA-signaling-via-NFKB pathways, which are onco-immunological pathways (Wang et al., 2009; De Simone et al., 2015; Mostofa et al., 2017; Silginer et al., 2017; Zhu et al., 2020).

Immune checkpoint blockade is a promising treatment for glioma patients. However, not all patients can benefit from it (Hodges et al., 2017). Therefore, we wonder whether this gene signature can be used as a predictive marker for the clinical response to immune checkpoint blocking therapy in LGG patients with epilepsy. The results showed that high-risk patients were more likely to benefit from anti-PD1 treatment. This may be related to their high expression of PD1/PD-L1, and the active IL6-STAT3 signaling and IFN-γ response pathways in high-risk patients can promote the expression of PD1/PD-L1 (Zhang et al., 2016; Qian et al., 2018; Litak et al., 2019). Also, a higher TMB was found in high-risk patients, and high TMB is currently considered to indicate a relatively better response rate to immunotherapy (Hellmann et al., 2018). We also found that high-risk patients might be more sensitive to TMZ therapy, which may be related to their active IFN pathway down-regulating MGMT level and increasing the sensitivity of glioma cells to TMZ (Shen et al., 2015).

In general, we established an immune-related gene signature, which can be used to predict the prognosis of LGG patients with epilepsy, and infer their immune infiltration status, response to immunotherapy and chemotherapy. Moreover, this study also deepens our understanding of the immune microenvironment in glioma. Of course, there are some limitations in this study. First of all, only TCGA data was used for analysis in this paper, and the sample size was relatively limited. It may be necessary to further expand the sample size and verify the results of this study in other databases. Secondly, due to the difficulty in obtaining clinical samples from LGG patients with epilepsy, no further *in vitro* experiments were conducted in this study to verify the results. In the end, the specific mechanisms of signature genes involved in glioma immunity need further exploration. The *p* values obtained from the analysis in this paper were summarized in Supplementary Table 7.

## Data Availability Statement

Publicly available datasets were analyzed in this study. This data can be found here: https://portal.gdc.cancer.gov/.

## Author Contributions

ZX and QC conceived and designed the research. QC and WD drafted and revised the manuscript. ZX, QC, and WD analyzed the data. KL, CL, SH, WY, BY, and HC acquired and interpreted the data. All authors contributed to the article and approved the submitted version.

## Conflict of Interest

The authors declare that the research was conducted in the absence of any commercial or financial relationships that could be construed as a potential conflict of interest. The handling editor declared a shared affiliation, though no other collaboration, with several of the authors WD, QC, and WY at the time of the review.

## Footnotes

1 https://portal.gdc.cancer.gov/

2 http://cibersort.stanford.edu/

3 https://www.cancerrxgene.org
