# Supplementary material for: Multi-Omics Data Integration Analysis of an Immune-Related Gene Signature in LGG Patients With Epilepsy
Source: Front Cell Dev Biol. 2021 Jul 16;9:686909. doi: 10.3389/fcell.2021.686909 (PMC8322853; doi:10.3389/fcell.2021.686909)
Supplement: Supplementary file 1 [file Table_1.docx]

Supplementary Material

# Supplementary Figures





**Supplementary Figure 1.** The process of sample screening.

#



**Supplementary Figure 2.** The 16 methylation sites most associated with expression in PDPN **(A)**, INA **(B)**, and ABCC3 **(C)** genes.





**Supplementary Figure 3.** Consensus clustering analysis. **(A)** CDF curves of k value from 2 to 10. **(B)** Relative change of the area under the CDF curve. **(C)** Heatmap of consensus matrix when k =2. **(D)** Principal component analysis. **(E)** Comparison of OS, DSS, and PFI between cluster 1 and cluster 2.





**Supplementary Figure 4.** Subgroup analysis of risk score. **(A)** WHO grade (grade II, grade III). **(B)** gender (male, female). **(C)** IDH status (mutant, wild type). **(D)**1p/19q status (non-codel, codel). **(E)** MGMT promotor status (unmethylated, methylated). **(F)** subtype (classical + mesenchymal, neural + proneural). **(G)** age (< 45, ≥ 45). *** p < 0.001.





**Supplementary Figure 5.** Comparison of gene mutation frequency between high- and low-risk groups.





**Supplementary Figure 6.** The biological pathways which had significant differences between high- and low-risk groups in GSVA.

**Supplementary Tables**

**Supplementary Table 1.** The characteristics of all included patients.

| **Characteristic** | **Non-Seizure** (n=176)^1^ | **Seizure** (n=297)^1^ | ***p*-value**^2^ |
| --- | --- | --- | --- |
| **Gender** |  |  | 0.4 |
| Female | 82(47%) | 127 (43%) |  |
| Male | 94 (53%) | 170 (57%) |  |
| **Age** | 42 (33, 54) | 40 (32, 52) | 0.4 |
| **Grade** |  |  | 0.086 |
| G2 | 77 (44%) | 155 (52%) |  |
| G3 | 98 (56%) | 142 (48%) |  |
| **IDH** |  |  | >0.9 |
| Mutant | 144 (82%) | 240 (82%) |  |
| WT | 32 (18%) | 54 (18%) |  |
| **MGMT** |  |  | 0.14 |
| Methylated | 140 (80%) | 252 (85%) |  |
| Unmethylated | 36 (20%) | 45 (15%) |  |

^1^ n (%); Median (IQR)

^2^ Pearson's Chi-squared test; Wilcoxon rank sum test

# Supplementary Table 2. Baseline data of LGG patients with epilepsy.

|  | **N** | **High immune score group n=113** | **Low immune score group n=184** | ***p*-value** |
| --- | --- | --- | --- | --- |
| **Age** | 297 | 39 (32,52) | 41 (33,52) | 0.8 |
| **Gender** | 297 |  |  | 0.7 |
| female |  | 46 (41%) | 81 (44%) |  |
| male |  | 67 (59%) | 103 (56%) |  |
| **IDH** | 294 |  |  | 0.002 |
| mutant |  | 80 (72%) | 160 (87%) |  |
| wild type |  | 31 (28%) | 23 (13%) |  |
| **pq** | 297 |  |  | < 0.001 |
| codel |  | 15 (13%) | 84 (46%) |  |
| noncodel |  | 98 (87%) | 100 (54%) |  |
| **MGMT** | 297 |  |  | 0.005 |
| methylated |  | 87 (77%) | 165 (90%) |  |
| unmethylated |  | 26 (23%) | 19 (10%) |  |

Statistics presented: median (IQR); n (%).

Statistical tests performed: Wilcoxon rank-sum test; chi-square test of independence.

**Supplementary Table 3-1.** Univariate Cox regression analysis of 22 immune cells.

|  | **Hazard.Ration** | **CI95** | ***p*-value** |
| --- | --- | --- | --- |
| **B_cells_naive** | 1.751 | 0-315828.142 | 0.928 |
| **B_cells_memory** | 0 | 0-0.364 | 0.036 |
| **Plasma_cells** | 0 | 0-5073035.609 | 0.471 |
| **T_cells_CD8** | 42.947 | 0.147-12529.121 | 0.194 |
| **T_cells_CD4_naive** | 0 | 0-5401209.145 | 0.238 |
| **T_cells_CD4_memory_resting** | 50.132 | 1.715-1465.334 | 0.023 |
| **T_cells_CD4_memory_activated** | Inf | 1.06041137519515e+174-Inf | < 0.001 |
| **T_cells_follicular_helper** | 0.026 | 0-84.863 | 0.376 |
| **T_cells_regulatory** | 1.4402E+13 | 263.389-7.87513655106823e+23 | 0.016 |
| **T_cells_gamma_delta** | 0 | 0-Inf | 0.683 |
| **NK_cells_resting** | 5.547 | 0.002-12514.773 | 0.664 |
| **NK_cells_activated** | 2.401 | 0.001-6822.234 | 0.829 |
| **Monocytes** | 0.014 | 0.001-0.265 | 0.004 |
| **Macrophages_M0** | 34.855 | 0.461-2633.133 | 0.108 |
| **Macrophages_M1** | 161.783 | 0.015-1758000.868 | 0.283 |
| **Macrophages_M2** | 2.307 | 0.413-12.886 | 0.341 |
| **Dendritic_cells_resting** | 0 | 0-8.92717482970183e+57 | 0.431 |
| **Dendritic_cells_activated** | 1.62E+35 | 2.14-1.22580167704711e+70 | 0.048 |
| **Mast_cells_resting** | 2.549 | 0.177-36.713 | 0.492 |
| **Mast_cells_activated** | 0.122 | 0.007-2.121 | 0.149 |
| **Eosinophils** | 0 | 0-325745155966.123 | 0.624 |
| **Neutrophils** | 56.557 | 0-766675294.788 | 0.630 |

**Supplementary Table 3-2.** Multivariate Cox regression analysis of 22 immune cells.

|  | **Hazard.Ratio** | **CI95** | ***p*-value** |
| --- | --- | --- | --- |
| **B_cells_memory** | 0 | 0-69.56 | 0.169 |
| **T_cells_CD4_memory_resting** | 1.43 | 0.02-97.32 | 0.868 |
| **T_cells_CD4_memory_activated** | Inf | 1.25493467071928e+178-Inf | 0 |
| **T_cells_regulatory** | 17244264106 | 0-1.25063439786483e+23 | 0.119 |
| **Monocytes** | 0.01 | 0-0.33 | 0.008 |
| **Dendritic_cells_activated** | 4.14E+43 | 44573681.86-3.85335417965622e+79 | 0.017 |

**Supplementary Table 4.** Univariate Cox regression analysis of risk score.

|  | **HR** | **HR.95L** | **HR.95H** | ***p*-value** |
| --- | --- | --- | --- | --- |
| **Risk** | 30.8371476 | 11.3535302 | 83.7562992 | < 0.001 |
| **Grade** | 3.27620679 | 1.83697343 | 5.84305181 | < 0.001 |
| **Age** | 1.06067828 | 1.03689618 | 1.08500585 | < 0.001 |
| **Gender** | 1.05973069 | 0.61539137 | 1.82490229 | 0.834 |
| **IDH** | 4.80134187 | 2.75145359 | 8.37843818 | < 0.001 |
| **Subtype** | 0.16904049 | 0.09669587 | 0.29551095 | < 0.001 |

**Supplementary Table 5.** Correlation between risk score and immune cell infiltration

| **symbol** | **Pearson correlation** | ***p*-value** |
| --- | --- | --- |
| **Macrophages** | 0.177 | 0.002 |
| **CD8 T cells** | 0.104 | 0.074 |
| **pDC** | 0.100 | 0.084 |
| **TReg** | 0.069 | 0.234 |
| **NK cells** | 0.047 | 0.421 |
| **Eosinophils** | -0.083 | 0.153 |
| **Mast cells** | -0.121 | 0.037 |
| **DC** | -0.122 | 0.036 |
| **Th17 cells** | -0.133 | 0.022 |
| **NK CD56dim cells** | -0.150 | 0.010 |
| **Tem** | -0.167 | 0.004 |
| **aDC** | -0.213 | < 0.001 |
| **Th1 cells** | -0.271 | < 0.001 |
| **B cells** | -0.283 | < 0.001 |
| **T helper cells** | -0.287 | < 0.001 |
| **iDC** | -0.291 | < 0.001 |
| **Cytotoxic cells** | -0.304 | < 0.001 |
| **Neutrophils** | -0.321 | < 0.001 |
| **Th2 cells** | -0.371 | < 0.001 |
| **Tgd** | -0.396 | < 0.001 |
| **Tcm** | -0.423 | < 0.001 |
| **T cells** | -0.504 | < 0.001 |
| **TFH** | -0.556 | < 0.001 |
| **NK CD56bright cells** | -0.598 | < 0.001 |

**Supplementary Table 6.** Correlation between risk score and inflammatory activities

| **symbol** | **Pearson correlation** | ***p*-value** |
| --- | --- | --- |
| **MHC_II** | 0.629 | < 0.001 |
| **STAT1** | 0.627 | < 0.001 |
| **LCK** | 0.623 | < 0.001 |
| **MHC_I** | 0.619 | < 0.001 |
| **HCK** | 0.551 | < 0.001 |
| **Interferon** | 0.508 | < 0.001 |
| **IgG** | -0.197 | < 0.001 |

**Supplementary Table 7.** P values of the analysis.

| **Analysis** | ***p*-value** |
| --- | --- |
| Survival analysis based on immune score in glioma patients with epilepsy | < 0.01 |
| Difference of signature genes expression | < 0.001 |
| Survival analysis based on signature genes expression | < 0.001 |
| Correlation analysis between methylation and expression levels | < 0.001 |
| Survival analysis based on consensus clustering | < 0.001 |
| Survival analysis based on risk model | < 0.001 |
| Correlation analysis between risk score and clinicopathological characteristic | < 0.001 |
| Correlation analysis between risk score and genomic mutation characteristic | < 0.001 |
| Survival analysis based on nomogram | < 0.001 |
| Correlation analysis between risk score and immune checkpoint expression | < 0.01 |
| Prediction of response to anti-PD1 therapy | 0.01 |
| Analysis of tumor mutation burden | < 0.01 |
| Prediction of response to temozolomide therapy | < 0.001 |
